# Optimizing Public Health Screening: Population-Specific BMI Thresholds for Targeted Body Composition Assessment in Hungary

**DOI:** 10.3390/nu18091410

**Published:** 2026-04-29

**Authors:** Tamas Jarecsny, Nadim Al-Muhanna, Dora Rebeka Fabian, Roland Kosik, Richard Schwab, Gergo Jozsef Szollosi, Laszlo Schandl, Gyula Tomasics, Eszter Melinda Pazmandi, Andras Folyovich, Ferenc Fazekas, Monika Fekete

**Affiliations:** 1 Department of Neurology and Stroke, Saint John’s Central Hospital of North Buda, 1125 Budapest, Hungary; jarecsny.tamas@janoskorhaz.hu (T.J.); nadim@janoskorhaz.hu (N.A.-M.); fabian.dora@janoskorhaz.hu (D.R.F.); fazekas.ferenc@stud.semmelweis.hu (F.F.); 2 Doctoral School, Semmelweis University, 1085 Budapest, Hungary; pazmandi.eszter.melinda@semmelweis.hu; 3 National Public Health and Pharmaceutical Center, 1097 Budapest, Hungary; kosik.roland@nngyk.gov.hu; 4 MiND Brain-Gut Center, 1125 Budapest, Hungary; mailbox@schwab.hu; 5 Faculty of Economics and Business, University of Debrecen, 4032 Debrecen, Hungary; szolgerjozs@gmail.com; 6 Department of Internal Medicine-Diabetology, Saint John’s Central Hospital of North Buda, 1125 Budapest, Hungary; schandl.laszlo@janoskorhaz.hu (L.S.); tomasics.gyula@janoskorhaz.hu (G.T.); 7 András Pető Faculty, Semmelweis University, 1125 Budapest, Hungary; 8 Kútvölgyi Outpatient Clinic, Saint John’s Central Hospital of North Buda, 1125 Budapest, Hungary; andras.folyovich@janoskorhaz.hu; 9 Cardiovascular Medicine Research Division, Doctoral College, Semmelweis University, 1085 Budapest, Hungary; 10 Health Services Management Training Centre, Semmelweis University, 1125 Budapest, Hungary; 11 Institute of Preventive Medicine and Public Health Faculty of Medicine, Semmelweis University, 1085 Budapest, Hungary; 12 Fodor Center for Prevention and Healthy Aging, Semmelweis University, 1085 Budapest, Hungary; 13 Health Sciences Division, Doctoral College, Semmelweis University, 1085 Budapest, Hungary

**Keywords:** nutritional status, body mass index, body composition, cardiometabolic risk, public health screening, cost-effectiveness, precision prevention, screening strategy

## Abstract

**Background**: Body mass index (BMI) is widely used as a proxy of nutritional status and related lifestyle risk patterns in public health, yet it does not capture body composition–related heterogeneity in cardiometabolic risk. Evidence on whether a more detailed body composition assessment improves population-level screening efficiency remains inconsistent, particularly in Central European populations. **Methods**: We conducted a cross-sectional analysis of 868 Hungarian adults participating in a nationwide mobile screening program. Locally weighted regression identified sex-specific BMI inflection points for cardiometabolic risk. Stratified receiver operating characteristic (ROC) analyses compared BMI with bioelectrical impedance-derived parameters across five outcomes. Cost- and time-effectiveness of scalable screening strategies were modeled at the population level. **Results**: Cardiometabolic risk increased at BMI levels below current WHO thresholds (females: 21.8–22.3 kg/m^2^; males: 23.8–24.3 kg/m^2^). Overall, body composition parameters did not outperform BMI in the full population. Subgroup-specific differences were observed, particularly among men with BMI 24–36 kg/m^2^ for atherosclerosis risk, suggesting limited and outcome-specific added value rather than broad superiority over BMI. Together, non-linear risk patterns, stratified performance, and population-level modeling converged on mid-range BMI intervals (females: 22–30 kg/m^2^; males: 24–30 kg/m^2^) as likely screening windows of phenotypic heterogeneity. Within these ranges, targeted InBody assessment may help refine risk assessment for selected individuals. A mixed screening strategy covering 52% of the population would cost 178.4% of BMI-only screening, while reducing throughput by 24.3%. **Conclusions**: Population-specific BMI thresholds may more accurately reflect early deviations in nutritional and cardiometabolic risk than current universal cutoffs. BMI remains a useful first-line marker, and body composition assessment may add complementary information in selected BMI ranges. Overall, these findings support a potentially useful, subgroup-specific screening approach, but the modeled cost and time trade-offs should be considered hypothesis-generating and require further validation.

## 1. Introduction

Cardiovascular disease and remain the leading causes of death and disability globally, with obesity serving as a major modifiable risk factor affecting over 1 billion adults worldwide [1,2,3,4,5]. Hungary faces particularly acute challenges: Recent data indicate that 72.5% of Hungarian adults are overweight or obese, substantially exceeding European Union averages (~55%) [6]. This high prevalence translates to a substantial healthcare burden: Cardiovascular and cerebrovascular disease account for nearly 50% of all-cause mortality in Hungary [7], and impose direct healthcare costs exceeding €3–4 billion annually [8].

Effective prevention requires efficient screening strategies, as costs are distributed across the population while benefits primarily accrue to high-risk individuals. This necessitates screening tools that balance sensitivity, specificity, cost, and implementability across diverse settings [9,10]. Body mass index (BMI) remains the standard due to its simplicity and portability, but it cannot distinguish adipose from lean mass, potentially misclassifying muscular or sarcopenic individuals [11,12]. Bioelectrical impedance analysis (BIA) theoretically improves risk stratification through body fat distribution [13,14,15], yet requires costly equipment (€3000–15,000), trained operators, and infrastructure that may be incompatible with resource-constrained mobile screening settings [16]. In public health, BMI and body composition are also widely interpreted as proxy markers of nutritional status, linking adiposity patterns to metabolic and inflammatory risk profiles [12,13,17].

Critical knowledge gaps persist due to conflicting international evidence on BIA: some studies report superiority over BMI for cardiometabolic outcomes [18,19,20], while others demonstrate comparable performance after accounting for statistical overlap [21,22,23]. Optimal BMI thresholds also vary across populations, with Asian studies consistently reporting lower cutoffs than current WHO standards [24,25,26,27,28,29,30]. Importantly, data from Central European populations remain scarce, particularly regarding the comparative performance of BIA and BMI in mobile screening settings. Moreover, clinical BMI ranges in which BIA provides meaningful incremental discrimination have not been systematically defined.

These gaps are particularly acute for Hungary, where preventive budgets remain constrained and mobile screening represents the primary mechanism for equitable access to underserved regions [31,32,33]. Universal BIA deployment proves economically unfeasible; selective implementation requires evidence defining high-value clinical subgroups where incremental diagnostic benefit justifies added costs. These findings are particularly relevant in the context of public health nutrition, where anthropometric and body composition measures are increasingly used to capture nutritional status and its metabolic consequences.

## 2. Aims

We analyzed data from 868 Hungarian adults participating in a nationwide mobile screening program to determine whether (1) BIA parameters provide incremental value over BMI for major cardiometabolic outcomes (hypertension, diabetes, hypercholesterolemia, hypertriglyceridemia, atherosclerosis), (2) sex- and outcome-specific performance differences exist, (3) specific BMI ranges can be identified where BIA adds discriminatory value, and (4) targeted screening strategies are cost-effective.

## 3. Materials and Methods

### 3.1. Study Design and Population

This cross-sectional analysis utilized InBody (Seoul, Republic of Korea) BIA measurements collected within the same screening framework as our published economic evaluation of BMI-based stroke PAF, conducted between June and August 2022 [6,32].

This nationwide mobile health program provides free, on-site preventive services, including oral cancer screening, gynecological examination, cardiometabolic risk assessment (blood pressure, serum glucose, lipid profile, electrocardiography), and body composition analysis by bioelectrical impedance.

For the present analysis, we included adults (age ≥ 30 years) who completed a core cardiometabolic module, defined as having: (1) reliable fasting laboratory measurements (glucose and lipids), (2) valid InBody body composition assessment, and (3) carotid duplex ultrasound.

Participants with missing or invalid measurements or contraindications to body composition assessment, such as pregnancy, pacemaker implantation, or major prostheses, were excluded. The final analytical sample comprised 868 participants with complete anthropometric and clinical data (547 women [63.0%], 321 men [37.0%]; median age 54.8 years, IQR 47.2–63.2; range 33–84 years).

The study followed STROBE reporting guidelines for observational studies.

### 3.2. Data Collection and Measurements

Structured questionnaires administered by trained staff collected comprehensive demographic and clinical information: age, biological sex, current medications, and presence of known chronic conditions.

Height was measured with a Soehnle ultrasonic stadiometer (height accuracy ± 0.5 cm; Soehnle Industrial Solutions, Backnang, Germany); fasting blood glucose, total cholesterol, and triglycerides were measured with a Multicare IN point-of-care analyzer and test strips (Biomedical Systems International, Arezzo, Italy); blood pressure was recorded by an integrated cuff device (MESI mTABLET ABI system, MESI Development of Medical Devices Ltd., Ljubljana, Slovenia); carotid duplex ultrasound was performed using a GE Versana Active 4D ultrasound system (GE HealthCare, Chicago, IL, USA).

Body composition was assessed using an InBody 270 bioelectrical impedance analyzer (InBody Inc., Seoul, South Korea). All measurements were taken with participants wearing light clothing and no shoes, preferably in the morning at least two hours after eating. Measurements included:

- Body mass index (BMI): Calculated as weight (kg)/height^2^ (m^2^); categorized as underweight (<18.5), normal weight (18.5–24.9), overweight (25.0–29.9), and obese (≥30.0) kg/m^2^ according to WHO guidelines.
- Body fat percentage (%): Proportion of total body weight composed of adipose tissue, expressed as a percentage of total body mass. Values < 25% in men and <35% in women are generally considered healthy [34].
- Skeletal muscle mass (kg): Total mass of striated muscle tissue excluding extracellular fluid and bone; represents functional muscular capacity and metabolic rate.
- Visceral fat level (1–20 proprietary scale): InBody algorithm-derived index estimating volume and mass of intra-abdominal (visceral) adipose tissue. Higher values (especially 15–20) indicate elevated visceral fat accumulation associated with increased metabolic risk. Lower values (<9) indicate minimal visceral fat deposition.
- Trunk fat ratio (0–400 proprietary InBody scale): Index of central adiposity calculated as (truncal fat mass/total fat mass) × 100, normalized to a proprietary InBody scale. The index ranges 0–400, with higher values (>300) indicating preferential truncal (central) rather than peripheral fat distribution, associated with increased metabolic risk.
- Total fat mass (kg): Absolute mass of adipose tissue throughout the body, calculated from BIA; used to assess total adiposity independent of body weight or BMI.

### 3.3. Missing Data and Data Quality

No missing BMI values occurred in the final dataset. All recorded measurements fell within physiologically plausible ranges; therefore, no observations were excluded from the analysis.

### 3.4. Definition of Outcome Variables

Cardiometabolic risk factors were defined as binary outcomes according to international guidelines. Hypertension was defined per the 2018 ESC/ESH guidelines as systolic blood pressure ≥ 140 mmHg and/or diastolic ≥ 90 mmHg or use of antihypertensive medication. Diabetes mellitus was diagnosed according to the 2021 ADA criteria: fasting plasma glucose ≥ 7.0 mmol/L or random glucose ≥ 11.1 mmol/L or use of antidiabetic medication. Dyslipidemia was defined by the 2019 ESC/EAS guidelines as total cholesterol > 5.2 mmol/L or triglycerides > 1.7 mmol/L. Atherosclerosis was diagnosed based on imaging-confirmed presence of carotid arterial plaque on ultrasound, defined as intima-media complex thickness > 1.5 mm or focal atherosclerotic lesions with acoustic shadowing or evidence in medical history.

### 3.5. Specification of Predictor Variables

The primary predictor was body mass index (BMI), calculated in kg/m^2^. Alternative body composition parameters included body fat percentage, visceral fat level (1–20 scale), trunk fat ratio, and muscle mass measured by the InBody 270 system. Age and sex were included in all multiple models as potential confounders.

### 3.6. Statistical Methods

Analyses were conducted in R version 4.2.1 (The R Foundation for Statistical Computing, Vienna, Austria) using the following packages: tidyverse (v1.3.2), survey (v4.1-1), epiR (v2.0.52), and boot (v1.3-28). Statistical significance was set at α = 0.05 (two-tailed). Descriptive statistics for continuous variables were presented as median ± interquartile range, and categorical variables as relative frequencies. Group comparisons were conducted using Mann–Whitney U tests for continuous variables and chi-square tests for categorical variables. Receiver operating characteristic (ROC) analysis was performed to evaluate discriminatory performance of each body composition parameter for predicting cardiometabolic risk outcomes, with area under the curve (AUC) values and 95% confidence intervals (CIs) calculated using DeLong’s method. All ROC analyses were performed separately for women and men, with formal statistical testing of sex × parameter interactions using multivariable logistic regression. Optimal cutoff values for each predictor were identified by maximizing the Youden index, balancing sensitivity and specificity for clinical utility. Paired ROC curve comparisons between BMI and alternative body composition parameters were conducted using DeLong tests to assess statistical significance of differences in AUC. Multiple testing correction was applied using Bonferroni method with adjusted α = 0.00065 (accounting for 77 comparisons across 5 outcomes × 5 parameters × 2 sexes + stratified analyses). Multivariable logistic regression models quantified independent associations between each predictor and cardiometabolic outcomes, adjusted for age and sex. Results were reported as adjusted odds ratios (AOR) with 95% CI per unit increase in each predictor. To identify non-linear associations between BMI and body composition parameters, locally weighted scatterplot smoothing (LOESS) regression was applied.

The analysis:

(1) Binned BMI data into 1 kg/m^2^ increment,

(2) For each BMI bin, calculated the prevalence of each individual cardiometabolic outcome (hypertension, diabetes, hypertriglyceridemia, atherosclerosis),

(3) Applied LOESS with parameters: span 0.3–0.75 (parameter-specific optimization), degree 2 (quadratic local fitting), tricube weight function,

(4) Identified inflection points where relationship linearity changed fundamentally.

Sex-specific optimal BMI ranges were defined as the BMI interval where the LOESS smoothed curve for individual outcomes reached lower prevalence values, reflecting lower disease risk. 95% CI around inflection points were calculated via bootstrap resampling (1000 iterations). To identify clinical circumstances where InBody parameters provide superior discriminatory performance despite lacking overall superiority, we performed stratified ROC analysis across predefined 3 kg/m^2^ BMI intervals. Participants were stratified into non-overlapping BMI groups: 18–21, 21–24, 24–27, 27–30, 30–33, 33–36, 36–39, and 39–42 kg/m^2^. Within each stratum, separate ROC curves were constructed for each body composition parameter and BMI to predict five cardiometabolic outcomes (hypertension, diabetes mellitus, hypercholesterolemia, hypertriglyceridemia, atherosclerosis). Statistical significance of differences between parameter-specific and BMI-based AUCs within each stratum was assessed using DeLong tests. A result was considered clinically significant if: (1) DeLong *p* < 0.05, and (2) the absolute AUC difference exceeded 0.10 (representing ≥ 10% relative improvement in discriminatory performance). Stratified analyses were performed separately for women and men. Results meeting both criteria are presented in the Section 4. While comprehensive stratified findings (including non-significant comparisons) are provided in Supplementary Table S1.

Screening throughput and labor costs were compared across BMI-only (2 min/patient), universal InBody (5 min/patient), and targeted InBody strategies (sex-specific BMI 24–30 for males and 22–30 for females). Costs were based on 2025 Hungarian wages (756,400 HUF/month; KSH) and the 2022 census population (7,921,212 adults aged 18 years or older). The mixed strategy was estimated as a weighted average using post-stratification weights (Supplementary Materials, Table S1). Detailed LOESS methodology and bootstrap confidence intervals are provided in Supplementary Materials S2. Complete stratified ROC results (77 comparisons) are presented in Supplementary Materials S3 (Tables S4 and S5). Cost modeling details are available in Supplementary Materials S4 (Tables S6–S11).

## 4. Results

### 4.1. Study Population Characteristics

The analysis included 868 participants (547 women [63.0%], 321 men [37.0%]; median age 54.8 years, IQR 47.2–63.2). The sex distribution reflected the composition of the underlying screening cohort and the requirement for complete data, rather than any deliberate sex-based sampling. Baseline characteristics and outcome prevalence by sex are presented in Table 1. Hypertension was the most prevalent outcome (484/868, 55.8%), followed by hypercholesterolemia (360/868, 41.5%). Men had significantly higher atherosclerosis prevalence (156/321, 48.6% vs. 177/547, 32.4%; *p* < 0.001) and hypertriglyceridemia (150/321, 46.7% vs. 236/547, 43.1%; *p* < 0.05). Median BMI was 27.6 kg/m^2^ (IQR 24.5–31.4), with women showing higher body fat percentage (37.2% vs. 27.1%; *p* < 0.001) and men higher muscle mass (36.7 kg vs. 25.0 kg; *p* < 0.001). Despite lower median BMI in women, higher body fat percentage translated to greater absolute fat mass (28.2 kg vs. 23.6 kg; *p* < 0.001).

### 4.2. Overall Predictive Performance

All predictors demonstrated moderate discrimination (AUC range 0.50–0.69, all *p* < 0.05 vs. chance). Overall predictive performance results are presented in Table 2. No InBody parameter significantly outperformed BMI in the overall population analysis (DeLong test, Bonferroni-corrected α = 0.00065), with one exception: body fat percentage for male atherosclerosis (AUC 0.656 [95% CI 0.597–0.716] vs. BMI 0.576 [0.514–0.639]; ΔAUC = 0.080, *p* = 0.001).

### 4.3. BMI Inflection Points and Risk Escalation Zones

LOESS regression identified sex-specific BMI inflection points where disease prevalence begins rising above baseline, all below WHO overweight (25 kg/m^2^) and obesity (30 kg/m^2^) cutoffs (Table 3). For women: hypertension 22.1 kg/m^2^, diabetes 21.8 kg/m^2^; for men: hypertension 24.0 kg/m^2^, diabetes 24.3 kg/m^2^, atherosclerosis 24.2 kg/m^2^ (all 95% CI ± 1.2 kg/m^2^ via 1000-iteration bootstrap).

The gap between inflection points (population-level risk emergence) and Youden-optimized cutoffs (clinical thresholds) defined risk escalation zones ranging 3.2–5.9 kg/m^2^ (Table 3). Narrowest zones occurred for female diabetes (3.2 kg/m^2^, BMI 21.8–25.0) and male diabetes (4.3 kg/m^2^), suggesting rapid risk acceleration requiring intensive monitoring. Widest zones occurred for hypertension (men 5.9 kg/m^2^, women 5.7 kg/m^2^), indicating gradual risk accumulation.

### 4.4. Stratified Analysis: Subgroup-Specific Differences in Discrimination

Stratified ROC analysis across 3 kg/m^2^ BMI intervals (18–42 kg/m^2^, non-overlapping) revealed seven comparisons in which InBody parameters were associated with better discrimination than BMI, using a priori criteria of DeLong *p* < 0.05 and ΔAUC > 0.10, as shown in Table 4 (see also Supplementary Materials S3, Tables S4 and S5). However, these results should be interpreted as exploratory, given the relatively small sample sizes in several strata and the potential for instability in subgroup-specific AUC estimates. The largest observed differences occurred mainly in men with BMI 24–36 kg/m^2^ predicting atherosclerosis (trunk fat ratio and body fat % improved AUCs by 0.17–0.35), plus lean men (BMI 18–21 kg/m^2^) for hypertension and hypercholesterolemia and obese women (BMI 36–39 kg/m^2^) for atherosclerosis.

### 4.5. Integrated Results: Complementary Screening Signals Across Mid-Range BMI Intervals

Overall ROC analysis across the full cohort showed only limited superiority of InBody over BMI, with a statistically significant advantage observed solely for body fat percentage in men predicting atherosclerosis (DeLong *p* < 0.05); no significant advantages were found for other parameters or outcomes.

LOESS analysis identified BMI escalation zones (women 22–27 kg/m^2^, men 24–29 kg/m^2^) where BMI and body composition parameters decoupled non-linearly, suggesting greater phenotypic heterogeneity.

Stratified ROC analyses further indicated that within some of these mid-range BMI intervals, InBody measures may provide additional discriminatory information for selected outcomes.

Taken together, these approaches provide complementary perspectives (LOESS identifies risk emergence, ROC quantifies discrimination, and stratified analyses explore heterogeneity) and converge on similar mid-range BMI intervals (women 22–30 kg/m^2^, men 24–30 kg/m^2^), supporting their interpretation as range of potential interest. In obese individuals (BMI ≥ 30 kg/m^2^), BMI alone is typically sufficient to trigger intervention in current practice and InBody functions primarily as a staging tool.

### 4.6. Modeled Implementation Scenario

Post-stratification weighting based on 2022 Hungarian census data and national overweight and obesity prevalence estimated that 51.9% of Hungarian adults (18+) would be targeted for InBody screening under a hypothetical sex-specific BMI algorithm (women 22–30 kg/m^2^, men 24–30 kg/m^2^) (Supplementary Materials S1, Table S2). This modeled scenario applies BMI-only assessment (2 min/patient) to low-risk strata and InBody assessment (5 min/patient) to high-phenotypic-heterogeneity zones. In this theoretical scenario, using Hungarian payment data, the mixed screening strategy would cost 178.4% of BMI-only screening, corresponding to a 78.4% increase in unit cost, while reducing throughput to approximately 75.7% of BMI-only capacity, representing a 24.3% decrease in screening capacity. Further studies are required to assess whether these cost and time trade-offs translate into meaningful clinical utility and to validate the real-world feasibility of the proposed screening strategy. Detailed cost and capacity calculations are provided in Supplementary Materials S4 (Tables S6–S11).

## 5. Discussion

### 5.1. Principal Findings

BMI demonstrated consistent, albeit moderate, discriminatory performance for hypertension, diabetes, hypertriglyceridemia, and atherosclerosis, supporting its role as a pragmatic first-line indicator in population-level risk assessment. In contrast, predictive performance for hypercholesterolemia was limited (AUC 0.50–0.53). Across the full cohort, InBody-derived parameters did not show overall superiority over BMI, with the exception of body fat percentage for male atherosclerosis (AUC 0.656 vs. 0.576; ΔAUC = 0.080, *p* = 0.001).

However, stratified analyses suggested heterogeneity across BMI ranges and sex-specific subgroups: several subsets were associated with higher discrimination when using InBody parameters, particularly among men with BMI 24–36 kg/m^2^ for atherosclerosis, as well as in selected lean and obese subpopulations. These findings indicate that body composition measures may add limited, outcome-specific information in certain groups, rather than providing broad superiority over BMI.

Importantly, both BMI and body composition parameters exhibited marked non-linear relationships with cardiometabolic outcomes. Concordant patterns in LOESS curves and ROC performance were consistent with a decoupling of BMI and underlying body composition within specific BMI strata, reflecting phenotypic heterogeneity. Sex-specific inflection points indicated the onset of risk acceleration at lower BMI values than current WHO thresholds (females: 21.8–22.3 kg/m^2^; males: 23.8–24.3 kg/m^2^), while higher Youden-derived cutoffs defined broader zones of progressive risk escalation.

Notably, these converging signals—non-linearity, stratified InBody performance, and inflection-based risk emergence—aligned within mid-range BMI intervals (22–30 kg/m^2^ in females, 24–30 kg/m^2^ in males). Within these ranges, body composition assessment may help refine risk assessment for some individuals, but the added value appears to be limited and context-dependent. The explored modeling scenario of a mixed screening strategy, which would direct InBody assessment to approximately 52% of the population, should therefore be viewed as preliminary and hypothesis-generating. Further investigation is needed to determine whether the modeled trade-offs in time and cost burden are justified by measurable clinical utility and real-world feasibility.

### 5.2. BMI Historical Context and Evolving Utility

Body mass index was developed by Adolphe Quetelet in 1832 as a population-level descriptor of “average man” body habitus (weight/height^2^), not as a clinical diagnostic tool [35]. Its association with all-cause mortality emerged in Western cohorts 1960–1980s, where U/J-shaped curves identified BMI 25–30 as optimal, forming WHO universal thresholds [36,37]. These mortality-derived cutoffs were validated primarily in white, middle-aged Western males during eras of relative nutritional stability [38,39]. Contemporary prevention targets morbidity (hypertension, diabetes, atherosclerosis), where inflection points marking risk emergence may precede mortality optima by 2–5 kg/m^2^ due to prolonged disease trajectories.

Sex-dimorphism (female risk emergence 1.5–2.5 kg/m^2^ earlier), ethnicity (Asian optima 22–23 vs. Western 27 kg/m^2^), and regional factors (Central European trunk fat patterning) necessitate context-specific thresholds [29,40,41]. These findings suggest that Hungarian inflection points (females 21.8–22.3, males 23.8–24.3 kg/m^2^) precede WHO standards, suggesting that morbidity-driven, population-tailored approaches to screening thresholds may warrant further investigation.

### 5.3. Comparison with Existing Literature

Sex-specific BMI inflection points (females 21.8–22.3 kg/m^2^, males 23.8–24.3 kg/m^2^) below WHO overweight threshold (25 kg/m^2^) align with Asian population studies consistently reporting optimal cardiometabolic cutoffs at 22–24 kg/m^2^ [41,42]. Multiple meta-analyses confirm lower BMI–risk relationships in East Asian cohorts, with hazard ratios for CVD accelerating at BMI 22–23 versus Western 27–30 kg/m^2^, supporting population-specific threshold adaptation for Central Europeans exhibiting intermediate adiposity-risk patterns [40,43].

BIA versus BMI findings mirror international heterogeneity. Pro-BIA studies report 5–15% AUC improvements for metabolic outcomes, particularly visceral fat prediction of incident diabetes (HR 2.8 per 1-SD increase) [44,45]. However, methodological critiques highlight statistical overlap after multivariable adjustment, with several large cohorts demonstrating equivalence once age, sex, and waist circumference controlled [46,47]. This study helps explain these discrepancies through stratified analysis, demonstrating InBody may add value within narrow BMI subgroups where phenotypic complexity peaks, thus suggesting limited, context-specific utility rather than universal superiority.

This represents an initial Central European contribution, as no prior analyses exist from post-socialist economies where CVD mortality remains 1.5–2.0 × Western rates despite convergence in risk factor profiles. Hungarian adults may exhibit distinct fat distribution patterns versus guideline-deriving populations, with higher trunk fat at equivalent BMI reflecting metabolic vulnerability not captured by WHO universal thresholds [48,49,50]. The identified female risk emergence 1.5–2.5 kg/m^2^ earlier than males across all outcomes suggests sex-dimorphic adiposity metabolism potentially unique to this genetic/environmental context, supporting the need for further regional studies.

Regarding cost-effectiveness, while selective screening optimizes screening economics, long-term CVD prevention through targeted weight loss demonstrates robust cost-effectiveness globally (ICERs £2000–12,000/QALY) [51,52]. Phenotype-stratified approaches could be explored as a potential means to address both immediate capacity constraints and long-term health economic considerations.

### 5.4. Clinical and Policy Implications

Current guidelines typically recommend intensive intervention at BMI ≥ 30 kg/m^2^ or BMI ≥ 27 kg/m^2^ in the presence of comorbidities; however, this threshold-based approach may overlook substantial phenotypic heterogeneity within “normal weight” and “overweight” categories [53].

Our findings suggest that BMI and body composition are not interchangeable measures and that body composition assessment may help distinguish clinically relevant subphenotypes, including “healthy overweight” individuals (characterized by lower central adiposity) and “metabolically obese normal weight” individuals with elevated visceral fat.

In this context, the identification of sex-specific inflection zones (females ≥ 22 kg/m^2^; males ≥ 24 kg/m^2^) may indicate BMI ranges that could be considered for further investigation in relation to earlier, targeted lifestyle interventions, even in the absence of overt cardiometabolic disease. Such an approach aligns with preventive strategies aiming to intervene prior to irreversible disease progression.

From a public health perspective, phenotype-informed screening approaches may have the potential to contribute to more efficient resource allocation by focusing additional assessments on subgroups in which phenotypic heterogeneity appears greater. This is particularly relevant in settings with constrained resources, where universal deployment of advanced body composition tools is not feasible.

International experiences support the feasibility of stratified prevention approaches. For example, workplace-based metabolic screening programs and targeted lifestyle interventions have demonstrated improvements in cardiometabolic outcomes across different populations [54,55,56,57,58,59,60]. While direct extrapolation should be made with caution, these models illustrate how phenotype-based risk stratification could be explored within prevention frameworks.

In the Hungarian context, a stepwise screening model could be considered, where BMI serves as an initial filter and targeted body composition assessment is applied within defined BMI ranges associated with higher phenotypic variability. Such an approach may complement existing primary care and occupational health structures, while maintaining scalability and cost-efficiency. Importantly, these findings should be interpreted as hypothesis-generating, and further prospective and interventional studies are required before clinical or guideline-level implementation can be recommended.

### 5.5. Limitations, Future Research, and Implementation Consideration

The cross-sectional design precludes causal inference between body composition parameters and cardiometabolic outcomes, although the observed associations are biologically plausible based on established obesity-related pathways.

Volunteer-based recruitment from mobile screening programs may introduce selection bias toward more health-conscious individuals, potentially underestimating population-level risk. Post-stratification weighting partially addressed age–sex imbalances but could not account for regional, socioeconomic, or educational factors. The mobile screening program primarily targeted underserved regions in Hungary, and the sample was not designed to ensure equal urban and rural representation; therefore, geographic generalizability to the national population may be limited.

Findings based on the InBody 270 device may not be directly generalizable to other BIA platforms or gold-standard methods such as DXA. In addition, discrimination for hypercholesterolemia remained limited across all predictors, restricting conclusions for lipid-related outcomes.

Several stratified BMI-sex-outcome subgroups, especially those in men, contained relatively small numbers of participants, which may have reduced statistical power and increased the uncertainty of subgroup-specific AUC estimates; therefore, these findings should be considered exploratory. In addition, statistical significance should not be equated with clinical usefulness, and prospective validation is needed to determine whether any observed differences have practical value.

Cost-effectiveness estimates were derived from modeled assumptions and may vary depending on real-world implementation factors, including infrastructure, case mix, and scaling effects.

Future research should prioritize prospective cohort validation of identified inflection points, direct comparison with alternative body composition methods, and pragmatic trials assessing whether phenotype-stratified screening improves clinical outcomes. Replication in other Central and Eastern European populations is also warranted.

## 6. Conclusions and Recommendations

Sex- and outcome-specific BMI inflection points in this Hungarian population were consistently lower than current WHO thresholds, indicating that population-specific cutoffs may warrant further investigation. BMI remains a practical first-line screening tool, and body composition assessment may add complementary information in selected BMI ranges. However, the added value appears limited and context-dependent. Future prospective studies are needed to determine whether these findings translate into improved clinical outcomes, and to clarify the practical role of phenotype-informed risk stratification in public health nutrition.

## Figures and Tables

**Table 1:** Baseline characteristics and outcome prevalence by sex.

Characteristic	Men (*n* = 321)	Women (*n* = 547)	All (*n* = 868)	*p*-Value
Age, median (IQR), years	55.2 (48.1–63.1)	54.2 (46.8–63.5)	54.8 (47.2–63.2)	0.420
BMI, median (IQR), kg/m^2^	28.2 (25.4–31.6)	27.3 (24.1–31.4)	27.6 (24.5–31.4)	0.080
Body fat %, median (IQR)	27.1 (22.6–31.5)	37.2 (32.2–42.7)	33.5 (27.0–40.2)	<0.001
Visceral fat level (1–20)	10.0 (8.0–14.0)	13.0 (9.0–17.0)	12.0 (9.0–16.0)	<0.001
Muscle mass, kg	36.7 (32.4–40.9)	25.0 (22.6–27.4)	27.6 (24.1–34.5)	<0.001
Fat mass, kg	23.6 (18.0–31.1)	28.2 (21.9–36.9)	26.6 (20.5–34.0)	<0.001
Trunk fat ratio	301.9 (230.7–387.8)	258.7 (192.2–332.5)	272.9 (202.1–352.0)	<0.001
Outcomes				
Hypertension, *n* (%)	202 (62.9)	282 (51.6)	484 (55.8)	<0.001
Diabetes mellitus, *n* (%)	42 (13.1)	77 (14.1)	119 (13.7)	0.69
Hypercholesterolemia, *n* (%)	117 (36.4)	243 (44.4)	360 (41.5)	0.03
Hypertriglyceridemia, *n* (%)	150 (46.7)	236 (43.1)	386 (44.5)	0.34
Atherosclerosis, *n* (%)	156 (48.6)	177 (32.4)	333 (38.4)	<0.001

Abbreviations: BMI, body mass index; IQR, interquartile range; *n*, sample size; %, percentage; kg, kilograms. Data are median (IQR) or *n* (%). *p*-values from Mann–Whitney U test (continuous) or chi-square test (categorical).

**Table 2:** DeLong test results: BMI vs. best InBody parameter.

Outcome	Sex	BMI AUC (95% CI)	Best Parameter	InBody AUC (95% CI)	ΔAUC	*p*-Value
Hypertension	Men	0.658 (0.598–0.717)	Trunk fat ratio	0.659 (0.599–0.719)	0.001	0.901
Hypertension	Women	0.690 (0.646–0.733)	Trunk fat ratio	0.679 (0.635–0.724)	−0.011	0.229
Atherosclerosis	Men	0.576 (0.514–0.639)	Body fat %	0.656 (0.597–0.716)	0.080	0.001
Atherosclerosis	Women	0.547 (0.495–0.599)	Body fat %	0.553 (0.501–0.605)	0.006	0.683
Diabetes mellitus	Men	0.656 (0.561–0.751)	Body fat %	0.648 (0.553–0.743)	−0.008	0.805
Diabetes mellitus	Women	0.638 (0.568–0.709)	Trunk fat ratio	0.617 (0.546–0.688)	−0.021	0.111
Hypertriglyceridemia	Men	0.676 (0.617–0.735)	Trunk fat ratio	0.664 (0.604–0.723)	−0.012	0.254
Hypertriglyceridemia	Women	0.573 (0.524–0.621)	Trunk fat ratio	0.575 (0.527–0.624)	0.002	0.779
Hypercholesterolemia	Men	0.526 (0.460–0.592)	Body fat %	0.540 (0.474–0.606)	0.014	0.529
Hypercholesterolemia	Women	0.500 (0.451–0.548)	Visceral fat level	0.508 (0.459–0.557)	0.008	0.487

Abbreviations: AUC, area under the curve; CI, confidence interval; BMI, body mass index; ΔAUC, difference in AUC (InBody − BMI). Statistically significant difference (*p* < 0.00065 Bonferroni-corrected) indicated in bold. Complete ROC results: Supplementary Table S4.

**Table 3:** LOESS inflection points and risk escalation zones.

Outcome	Sex	Inflection Point (kg/m^2^)	Youden Cutoff (kg/m^2^)	Zone Width (kg/m^2^)
Hypertension	Men	24.0	29.9	5.9
	Women	22.1	27.8	5.7
Diabetes mellitus	Men	24.3	28.6	4.3
	Women	21.8	25.0	3.2
Hypertriglyceridemia	Men	23.8	29.2	5.4
	Women	22.3	26.4	4.1
Atherosclerosis	Men	24.2	28.8	4.6
	Women	22.0	26.8	4.8

Inflection points represent population-level risk emergence; Youden cutoffs represent optimal clinical thresholds balancing sensitivity/specificity. Zone width indicates BMI range of progressive risk escalation. LOESS curves are presented in Supplementary Materials S2 (Figure S1), and sex-specific inflection points with 95% confidence intervals are provided in Supplementary Materials S2 (Table S3).

**Table 4:** Stratified differences favoring InBody (DeLong *p* < 0.05, ΔAUC > 0.10).

Sex	BMI Range (kg/m^2^)	Outcome	Parameter	N/*n*	BMI AUC (95% CI)	InBody AUC (95% CI)	ΔAUC	*p*-Value
Male	18–21	Hypertension	Visceral fat	24/11	0.438 (0.109–0.766)	0.838 (0.588–1.000)	0.400	0.048
Male	18–21	Hypercholesterolemia	Muscle mass	24/7	0.333 (0.026–0.641)	0.714 (0.427–1.000)	0.381	0.043
Male	27–30	Atherosclerosis	Trunk fat ratio	82/46	0.499 (0.374–0.624)	0.666 (0.547–0.784)	0.167	0.018
Male	27–30	Atherosclerosis	Body fat %	82/46	0.499 (0.374–0.624)	0.662 (0.543–0.782)	0.163	0.044
Male	33–36	Atherosclerosis	Body fat %	36/23	0.506 (0.274–0.739)	0.857 (0.708–1.000)	0.351	0.029
Male	33–36	Atherosclerosis	Trunk fat ratio	36/23	0.506 (0.274–0.739)	0.818 (0.651–0.985)	0.312	0.037
Female	36–39	Atherosclerosis	Trunk fat ratio	29/19	0.382 (0.167–0.597)	0.648 (0.439–0.858)	0.266	0.046

Abbreviations: BMI, body mass index; N = sample size, *n* = outcome positive cases; AUC, area under the curve; CI, confidence interval; ΔAUC, difference in AUC (InBody − BMI). BMI ranges defined as left-closed, right-open intervals (e.g., 24–27 = BMI ≥ 24 and <27 kg/m^2^). Only cases meeting both significance (*p* < 0.05) and clinical relevance (ΔAUC > 0.10) thresholds shown. Complete stratified results are provided in Supplementary Materials S3 (Tables S4 and S5), and ROC curves for significant comparisons are presented in Supplementary Materials S3 (Figure S2).

## Data Availability

The data that support the findings of this study are available from the corresponding author upon reasonable request.

## Supplementary Materials

The following supporting information can be downloaded at: https://www.mdpi.com/article/10.3390/nu18091410/s1, Supplementary Materials S1: Post-stratification weighting. Table S1. Post-stratification weights by age group and sex. Table S2. Population eligible for targeted InBody screening based on sex-specific BMI inflection points. Supplementary Materials S2: Inflection point analysis. Figure S1. LOESS curves (BMI vs. outcome prevalence, sex-specific). Table S3. Sex-specific BMI inflection points. Supplementary Materials S3: ROC analysis results. Table S4. Predictive performance of body composition parameters by sex. Table S5. Significant stratified InBody superiority (DeLong *p* < 0.05, ΔAUC > 0.10). Figure S2. ROC curves for significant comparisons. Supplementary Materials S4: Screening time and cost-efficiency of BMI versus InBody measurement. Table S6. Cost per measurement. Table S7. Screening capacity per hour. Table S8. Cost and capacity comparison. Table S9. Population-level screening costs. Table S10. Wage sensitivity analysis (±20%). Table S11. Measurement time sensitivity (±20%).
