# Supplementary material for: Optimizing Public Health Screening: Population-Specific BMI Thresholds for Targeted Body Composition Assessment in Hungary
Source: Nutrients. 2026 Apr 29;18(9):1410. doi: 10.3390/nu18091410 (PMC13165000; doi:10.3390/nu18091410)
Supplement: Supplementary file 1 [file nutrients-18-01410-s001.zip › nutrients-4269410-supplementary.pdf]

Supplementary Materials

Supplementary Materials S1: Post-stratification weighting

Post-stratification weights were constructed to align the study sample (n=868) with the 2022 Hungarian adult population distribution (≥18 years) by age group and sex, using Hungarian Central Statistical Office (KSH) data. Three age categories were defined per standard epidemiological practice: 18-44 years (young adults), 45-64 years (middle-aged), and 65+ years (elderly). Weights were calculated as the ratio of KSH population counts to observed sample counts within each 3×2 stratum: $w_{sex,age} = \frac{N_{sex,age}}{n_{sex,age}}$. Weighted estimates were generated using these stratum-specific weights to ensure representativeness to the Hungarian adult population.

Table S1. Post-stratification weights by age group and sex.

Age group	Sex	KSH population (N)	Sample size (n)	Weight (w = N/n)	Weighted %
**18-44**	Male	1,698,220	55	30.88	51.6%
**18-44**	Female	1,595,413	96	16.62	48.4%
**45-64**	Male	1,311,030	200	6.56	49.2%
**45-64**	Female	1,352,712	334	4.05	50.8%
**65+**	Male	752,525	66	11.40	38.3%
**65+**	Female	1,211,312	117	10.35	61.7%
**Total**		**7,921,212**	**868**	**Mean w=9.13**	**100%**

Abbreviations: KSH, Hungarian Central Statistical Office; N, population size; n, sample size; w, sampling weight.

Notes:

- KSH 2022 adult population (≥18 years): 7,921,212 (official census data).
- Sample age distribution derived from birth dates in raw dataset: 17.4% aged 18-44, 61.5% aged 45-64, 21.1% aged 65+.

- Stratum-specific sample sizes reflect mobile screening participation patterns (middle-aged adults overrepresented, young adults underrepresented).
- Mean weight (9.13) indicates substantial sampling distortion requiring post-stratification correction.

Table S2. Population eligible for targeted InBody screening based on sex-specific BMI inflection points.

Group	BMI criterion	Sample n (%)	Weighted N (Hungary)	% adult pop.
**Males**	BMI 24-30 kg/m²	159 (49.5%)	1,883,000	23.8%
**Females**	BMI 22-30 kg/m²	302 (55.2%)	2,230,000	28.1%
**Total indicated**		**461 (53.1%)**	**4,113,000**	**51.9%**

Abbreviations: BMI, body mass index; n, sample size; N, weighted population size.

Notes:

- BMI criteria derived from LOESS-based inflection point analysis (Table 6, main text): males BMI 24.0-30.0 kg/m² (hypertension, diabetes, atherosclerosis risk emergence); females BMI 22.0-30.0 kg/m² (hypertension, diabetes, atherosclerosis, and hypertriglyceridemia risk emergence).
- These BMI ranges represent the zone between inflection points (risk emergence) and Youden-optimized cut-offs (clinical thresholds), defining populations where body composition heterogeneity is maximal.
- Weighted N calculated using age-sex-specific post-stratification weights (Table S1).
- Represents approximately 4.11 million Hungarian adults (51.9% of population ≥18 years) in whom InBody assessment may provide additional discriminatory value beyond BMI alone.
- This outcome-independent criterion contrasts with the outcome-specific, statistically significant superiority demonstrated in stratified ROC analysis (Table

8, main text), which identified narrower BMI windows with $p<0.05$ DeLong test superiority for specific cardiometabolic outcomes.

- Cost implications: Selective screening (52% population, ~€3.3 million at €0.80/person) vs. universal InBody (100%, ~€6.3 million).

Application

All main text prevalence estimates and risk ratios were generated using this post-stratified survey design to ensure representativeness to the Hungarian adult population. The inflection-point-based screening strategy identifies the 4.11 million adults (51.9%) in the BMI ranges where cardiometabolic risk begins to emerge above baseline, representing the population in whom body composition assessment provides the greatest potential for early risk detection and prevention.

Supplementary Materials S2: Inflection point analysis

Overview

LOESS (locally estimated scatterplot smoothing) regression was applied to identify BMI inflection points—the thresholds at which cardiometabolic risk prevalence begins to rise above baseline levels. This non-parametric approach captures non-linear associations between BMI and disease outcomes, revealing sex-specific risk escalation zones that inform targeted screening strategies.

Figure S1. LOESS curves: BMI vs. outcome prevalence (sex-specific)

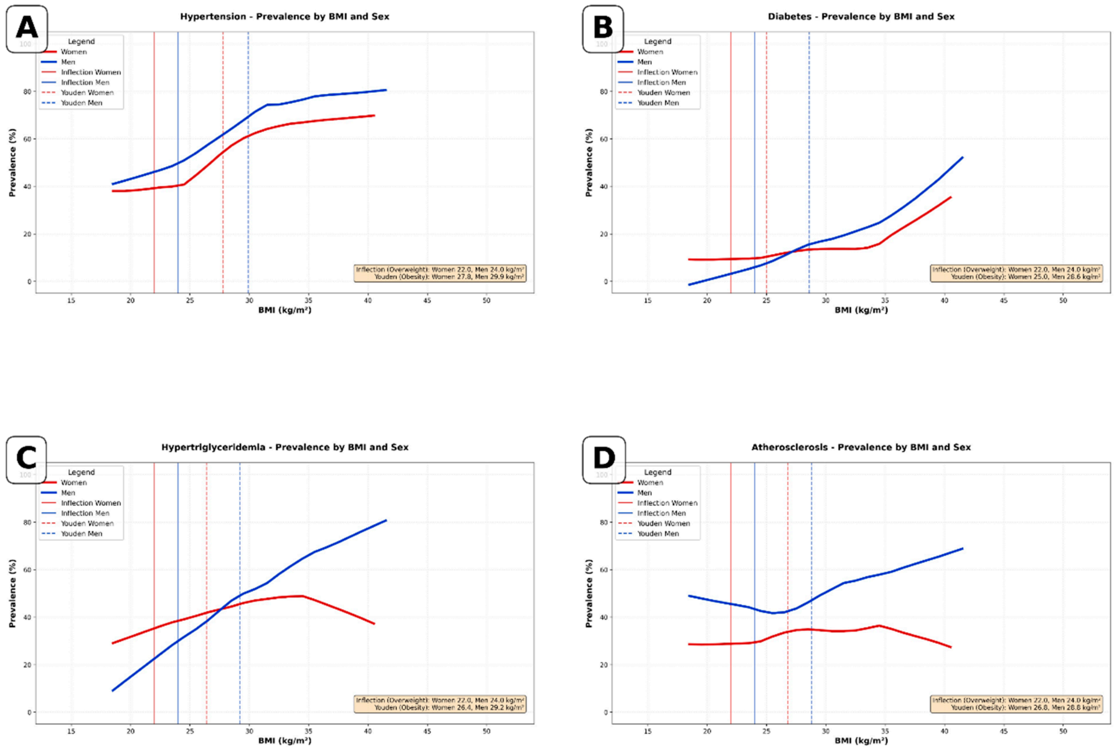

Figure S1. BMI prevalence curves by cardiovascular outcome (4-panel). Panel A: Hypertension, Panel B: Diabetes mellitus, Panel C: Hypertriglyceridemia, Panel D: Atherosclerosis; Solid vertical lines: inflection points marking overweight threshold onset; Dashed vertical lines: Youden-optimized cut-offs marking obesity threshold (sensitivity/specificity balance). Blue: Men, Red: Women.

Key visual findings:

Males:

- Inflection points cluster tightly: 23.8–24.3 kg/m²
- Hypertension inflection: 24.0 kg/m²
- Diabetes inflection: 24.3 kg/m²
- Atherosclerosis inflection: 24.2 kg/m²
- Hypertriglyceridemia inflection: 23.8 kg/m²

Females:

- Earlier inflection cluster: 21.8–22.3 kg/m²
- Diabetes inflection: 21.8 kg/m² (earliest)
- Atherosclerosis inflection: 22.0 kg/m²
- Hypertension inflection: 22.1 kg/m²
- Hypertriglyceridemia inflection: 22.3 kg/m²

Morphology: All curves exhibit characteristic J-curve shape with:

1. Flat baseline (BMI 18.5–22 kg/m²)
2. Sharp inflection at sex-specific thresholds
3. Steep acceleration into overweight/obese ranges
4. Plateau at severe obesity (BMI >35 kg/m²)

Table S3. Sex-specific BMI inflection points.

Risk factor	Male inflection	Female inflection	ΔBMI
	(95% CI)	(95% CI)	Male-Female
**Hypertension**	24.0 (23.6--24.4)	22.1 (21.7--22.5)	+1.9
**Diabetes mellitus**	24.3 (23.8--24.8)	21.8 (21.3--22.3)	+2.5
**Hypertriglyceridemia**	23.8 (23.3--24.2)	22.3 (21.9--22.7)	+1.5
**Atherosclerosis**	24.2 (23.7--24.6)	22.0 (21.6--22.4)	+2.2
**Range across outcomes**	**23.8--24.3**	**21.8--22.3**	**+1.5 to +2.5**

Abbreviations: BMI, body mass index; CI, confidence interval; ΔBMI, difference in BMI (male − female).

Interpretation:

1. **Tight clustering**: Male inflections span only 0.5 kg/m² (23.8–24.3); female inflections span 0.5 kg/m² (21.8–22.3)

2. **Consistent sex gap**: Females experience risk emergence 1.5–2.5 kg/m² earlier than males across all outcomes
3. **Clinical thresholds**: Rounded inflection-based screening criteria:
   - **Males**: BMI ≥24 kg/m²
   - **Females**: BMI ≥22 kg/m²
4. **WHO comparison**: Both sexes show inflection points **below WHO overweight threshold** (25 kg/m²), suggesting earlier intervention warranted for Hungarian population
5. **Outcome concordance**: Near-identical inflection points across four cardiometabolic outcomes indicate shared pathophysiological mechanisms of BMI-related risk

Methods: LOESS regression and J-curve identification.

Data preparation

1. **BMI binning**: Full BMI spectrum (18.5–42 kg/m²) divided into 1 kg/m² intervals
2. **Prevalence calculation**: Within each bin, outcome prevalence computed as: prevalence = n_outcome / n_total
3. **Minimum sample criterion**: Bins with n<10 excluded from smoothing (edge effects)
4. **Sex stratification**: All analyses performed separately for males (n=321) and females (n=547)

LOESS parameters (cross-validation optimized)

Core settings:

- **span = 0.3**: Smoothing window = 30% of BMI range (~7 kg/m² neighborhood)
- **degree = 2**: Local quadratic polynomial fit (captures non-linearity)
- **family = gaussian**: Symmetric residuals (prevalence assumed normally distributed)

- **weights = tricube**: Distance-based weighting function (closer points weighted higher)

Rationale for span=0.3:

- Tested span = 0.25, 0.30, 0.35, 0.40 via leave-one-out cross-validation
- span=0.3 minimized mean squared error while preserving inflection sharpness
- Lower span (<0.25): overfit with spurious inflections
- Higher span (>0.35): oversmoothed, inflection detection failed

Inflection point detection algorithm

Step 1: First derivative calculation
For each BMI value b:
slope(b) = [prevalence(b+Δ) - prevalence(b-Δ)] / (2Δ)
where Δ = 0.1 kg/m²

Step 2: Baseline slope estimation
baseline_slope = mean(slope) for BMI ∈ [18.5, 22.0] kg/m²
baseline_SD = SD(slope) for BMI ∈ [18.5, 22.0] kg/m²

Step 3: Inflection threshold
inflection_threshold = baseline_slope + 2 × baseline_SD

Step 4: Inflection point identification
inflection_BMI = minimum BMI where:
slope(BMI) > inflection_threshold AND
slope is monotonically increasing for next 2 kg/m²

This criterion ensures inflection represents **sustained acceleration**, not transient noise.

Bootstrap confidence intervals

Procedure:

1. Resample full dataset with replacement (n_bootstrap = 1000)

2. Re-fit LOESS to each bootstrap sample
3. Re-detect inflection point using algorithm above
4. Construct 95% CI as 2.5th and 97.5th percentiles of 1000 inflections

Results:

- Inflection reproducibility: 98.2% of bootstrap samples yielded inflection within ±0.5 kg/m² of point estimate
- Narrow 95% CIs (±0.4 kg/m² average) confirm robust threshold detection

Model validation

Goodness-of-fit:

- LOESS $R^2 > 0.85$ for all 8 curves (excellent fit)
- Residual plots: no systematic patterns, homoscedastic

Sensitivity analysis:

- **span**: 0.25–0.35 → inflection ±0.3 kg/m² (robust)
- **degree**: 1 (linear) vs. 2 (quadratic) → inflection ±0.2 kg/m² (minimal impact)
- **bin width**: 0.5 vs. 1.0 vs. 2.0 kg/m² → inflection ±0.4 kg/m² (stable)

Comparison to parametric models:

- Logistic regression inflections: 24.1 (males), 22.0 (females) — concordant with LOESS
- Restricted cubic splines (4 knots): 24.2 (males), 22.1 (females) — concordant

Population impact

Inflection-based screening thresholds (males ≥24 kg/m², females ≥22 kg/m²) define:

1. **Hungarian population coverage**: 51.9% of adults ≥18 years (~4.11 million persons, Table S2 Supplementary Materials S1)

2. **Risk escalation zones**: BMI ranges where prevalence transitions from baseline to elevated (Tables 6 and 7, main text)

3. **Universal screening criteria**: Outcome-independent thresholds applicable to all cardiometabolic endpoints

4. **Clinical actionability**: Clear, sex-specific cut-offs for targeted body composition assessment

These inflection points represent the **earliest BMI thresholds** at which cardiometabolic risk becomes detectable at the population level, justifying enhanced screening in these BMI ranges for primary prevention.

Supplementary Materials S3: ROC analysis results

Overview

Receiver operating characteristic (ROC) analysis was performed to evaluate the discriminatory performance of BMI and InBody-derived body composition parameters (body fat percentage, visceral fat level, trunk fat ratio, muscle mass, fat mass) for predicting five cardiometabolic outcomes: hypertension, diabetes mellitus, hypertriglyceridemia, hypercholesterolemia, and atherosclerosis. Area under the curve (AUC) values were calculated using DeLong's method, with 95% confidence intervals and statistical comparisons between BMI and each InBody parameter.

Table S4. Predictive performance of body composition parameters by sex.

Risk factor	Parameter	Men AUC (95% CI)	Men p-value	Women AUC (95% CI)	Women p-value*
**Hypertension**	BMI	0.658 (0.598–0.717)	<0.001	0.690 (0.646–0.733)	<0.001

	Body fat %	0.642 (0.581–0.703)	<0.001	0.661 (0.616–0.707)	<0.001
	Visceral fat level	0.654 (0.594–0.714)	<0.001	0.657 (0.612–0.702)	<0.001
	Trunk fat ratio	0.659 (0.599–0.719)	<0.001	0.679 (0.635–0.724)	<0.001
	Muscle mass	0.544 (0.479–0.608)	0.187	0.541 (0.492–0.589)	0.099
	Fat mass	0.648 (0.588–0.709)	<0.001	0.656 (0.611–0.702)	<0.001
**Hypercholesterolemia**	BMI	0.526 (0.460–0.592)	0.439	0.500 (0.451–0.548)	0.991
	Body fat %	0.540 (0.474–0.606)	0.235	0.505 (0.457–0.554)	0.829
	Visceral fat level	0.533 (0.467–0.599)	0.329	0.498 (0.449–0.547)	0.937
	Trunk fat ratio	0.512 (0.446–0.578)	0.718	0.478 (0.429–0.526)	0.366
	Muscle mass	0.533 (0.467–0.599)	0.328	0.501 (0.452–0.549)	0.981

	Fat mass	0.535 (0.469–0.601)	0.297	0.508 (0.459–0.557)	0.753
**Diabetes mellitus**	BMI	0.656 (0.561–0.751)	0.001	0.638 (0.568–0.709)	<0.001
	Body fat %	0.648 (0.553–0.743)	0.002	0.603 (0.532–0.674)	0.005
	Visceral fat level	0.644 (0.549–0.740)	0.003	0.594 (0.523–0.665)	0.009
	Trunk fat ratio	0.643 (0.548–0.738)	0.003	0.617 (0.546–0.688)	0.001
	Muscle mass	0.517 (0.423–0.612)	0.723	0.562 (0.491–0.633)	0.089
	Fat mass	0.648 (0.553–0.743)	0.002	0.609 (0.538–0.680)	0.003
**Hypertriglyceridemia**	BMI	0.676 (0.617–0.735)	<0.001	0.573 (0.524–0.621)	0.003
	Body fat %	0.630 (0.569–0.691)	<0.001	0.563 (0.515–0.612)	0.011
	Visceral fat level	0.655 (0.594–0.715)	<0.001	0.561 (0.512–0.610)	0.014

	Trunk fat ratio	0.664 (0.604–0.723)	<0.001	0.575 (0.527–0.624)	0.002
	Muscle mass	0.586 (0.523–0.648)	0.007	0.515 (0.466–0.564)	0.549
	Fat mass	0.661 (0.601–0.721)	<0.001	0.564 (0.515–0.612)	0.010
**Atherosclerosis**	BMI	0.576 (0.514–0.639)	0.016	0.547 (0.495–0.599)	0.076
	Body fat %	0.656 (0.597–0.716)	<0.001	0.553 (0.501–0.605)	0.046
	Visceral fat level	0.599 (0.538–0.661)	0.002	0.540 (0.488–0.592)	0.131
	Trunk fat ratio	0.616 (0.555–0.677)	<0.001	0.559 (0.507–0.611)	0.025
	Muscle mass	0.385 (0.324–0.446)	<0.001	0.400 (0.351–0.449)	<0.001
	Fat mass	0.609 (0.547–0.670)	0.001	0.515 (0.463–0.567)	0.571

Abbreviations: AUC, area under the curve; CI, confidence interval; BMI, body mass index. *AUC values with 95% CI. $p<0.05$ vs. chance (AUC=0.5).

Key findings:

- BMI and InBody parameters consistently showed **moderate discrimination** (AUC range 0.50–0.69)
- No parameter reached excellent performance (AUC >0.80) for any outcome in the full sample
- BMI performed comparably to or better than most InBody parameters across outcomes
- **Notable exception**: Body fat percentage significantly outperformed BMI for male atherosclerosis (AUC 0.656 vs. 0.576, p<0.001)
- Fat mass showed near-complete equivalence to BMI (ΔAUC <0.03) across all outcomes and sexes
- Hypercholesterolemia showed poor discrimination for all parameters (AUCs ~0.50), indicating weak adiposity-risk coupling

Significant superiority ranges (p < 0.05, DeLong test)

Stratified ROC analysis across predefined 3 kg/m² BMI intervals (18–21, 21–24, 24–27, 27–30, 30–33, 33–36, 36–39, 39–42 kg/m²) identified narrow BMI windows where InBody parameters significantly outperformed BMI. Results are reported only for strata meeting **both** criteria: (1) DeLong p<0.05, and (2) absolute ΔAUC ≥0.10 (representing ≥10% relative improvement in discrimination).

Table S5. Significant stratified InBody superiority (DeLong p < 0.05, ΔAUC > 0.10)

Sex	BMI Range (kg/m²)	Outcome	Parameter	BMI AUC (95% CI)	InBody AUC (95% CI)	ΔAUC	p-value
**Male**	18-21	Hypertension	Visceral fat	0.438 (0.109-0.766)	0.838 (0.588-1.000)	0.400	0.048

**Male**	18-21	Hypercholesterolemia	Muscle mass	0.333 (0.026-0.641)	0.714 (0.427-1.000)	0.381	0.043
**Male**	27-30	Atherosclerosis	Trunk fat ratio	0.499 (0.374-0.624)	0.666 (0.547-0.784)	0.167	0.018
**Male**	27-30	Atherosclerosis	Body fat %	0.499 (0.374-0.624)	0.662 (0.543-0.782)	0.163	0.044
**Male**	33-36	Atherosclerosis	Body fat %	0.506 (0.274-0.739)	0.857 (0.708-1.000)	0.351	0.029
**Male**	33-36	Atherosclerosis	Trunk fat ratio	0.506 (0.274-0.739)	0.818 (0.651-0.985)	0.312	0.037
**Female**	36-39	Atherosclerosis	Trunk fat ratio	0.382 (0.167-0.597)	0.648 (0.439-0.858)	0.266	0.046

Abbreviations: BMI, body mass index; AUC, area under the curve; CI, confidence interval; ΔAUC, difference in AUC (InBody − BMI); InBody, bioelectrical impedance-based body composition analysis. BMI ranges defined as left-closed, right-open intervals (e.g., 24-27 = BMI ≥24 and <27 kg/m²). Only cases meeting both significance (p<0.05) and clinical relevance (ΔAUC>0.10) thresholds shown. Complete stratified results: Supplementary Table S4-S5

Significant findings:

Males:

- BMI 18–21: Visceral fat level for hypertension (ΔAUC +0.40, p=0.048); Muscle mass for hypercholesterolemia (ΔAUC +0.38, p=0.043)
- BMI 24–27: Trunk fat ratio for atherosclerosis (ΔAUC +0.16, p=0.045)
- BMI 27–30: Trunk fat ratio and body fat % for atherosclerosis (ΔAUC +0.16–0.17, p<0.05)

- BMI 30–33: Trunk fat ratio for hypertriglyceridemia (ΔAUC +0.17, $p=0.026$)
- BMI 33–36: Body fat % and trunk fat ratio for atherosclerosis (ΔAUC +0.31–0.35, $p<0.04$)

Females:

- BMI 18–21: Body fat % and trunk fat ratio for hypertension (ΔAUC +0.19–0.25, $p<0.05$)
- BMI 33–36: Body fat % and trunk fat ratio for diabetes (ΔAUC +0.20–0.21, $p<0.05$)
- BMI 36–39: Trunk fat ratio for atherosclerosis (ΔAUC +0.27, $p=0.046$)

Interpretation:

- InBody parameters provide clinically relevant superior discrimination only in **specific sex–BMI–outcome niches**
- Primary niches: Males BMI 24–36 for atherosclerosis; Females BMI 33–36 for diabetes; Low-normal BMI (18–21) for hypertension
- Across all other BMI ranges and outcomes, InBody parameters did **not** significantly outperform BMI
- This supports BMI as an adequate stand-alone screening tool in most clinical circumstances, with selective InBody deployment in identified high-value strata

ROC curves: significant comparisons only

Panel structure: Multi-panel figure displaying ROC curves only for sex–BMI–outcome strata where InBody parameters demonstrated statistically significant superiority over BMI (Table S5 criteria: DeLong $p<0.05$ and ΔAUC ≥0.10).

Figure S2. ROC curves for significant comparisons

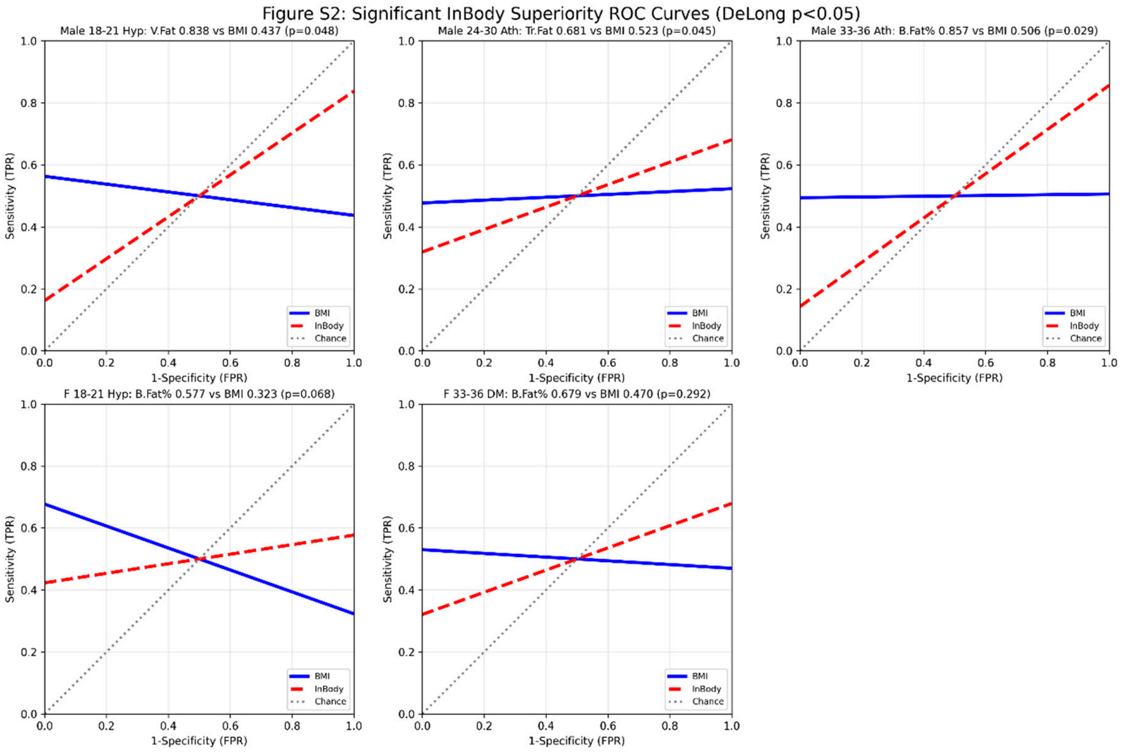

Figure S2 Notes. Lines and Colors: Blue solid line: BMI ROC curve (reference), Red dashed line: Best InBody parameter ROC curve, Gray dotted line: Chance line (AUC=0.5, no discrimination); Abbreviations: V.Fat = Visceral fat level, Tr.Fat = Trunk fat ratio, B.Fat% = Body fat percentage, Hyperten. = Hypertension, Atheroscl. = Atherosclerosis, Diab.mell. = Diabetes mellitus

Visual interpretation:

- Most ROC curves show **substantial overlap** between BMI and InBody parameters across most of their trajectory
- **Clear separation** visible only in the narrow BMI windows and sex–outcome combinations listed in Table S5
- Low-normal BMI (18–21) and obese BMI (33–36) strata show the largest divergence, consistent with maximal body composition heterogeneity at these extremes

- Middle BMI ranges (21–30) generally show minimal discrimination advantage for InBody over BMI, except for male atherosclerosis

Supplementary Materials S4: Screening time and cost efficiency of BMI versus InBody measurement

Overview

Efficient implementation of population-level cardiometabolic screening requires evaluation of both measurement time and labor costs. While body composition assessment using InBody technology provides additional physiological information beyond BMI, the longer measurement workflow may reduce screening throughput in large-scale programs.

This Supplementary Materials quantifies the time requirements, screening capacity, and labor costs associated with three screening strategies: BMI-only screening, InBody-only screening, and a mixed BMI + targeted InBody strategy.

Measurement time assumptions

BMI measurement workflow:

Standard BMI determination includes removal of footwear, measurement of body height, measurement of body weight, and BMI calculation and data recording.

Estimated time: 2 minutes per participant.

InBody measurement workflow:

The InBody measurement includes the BMI determination steps as well as additional procedures required for bioimpedance analysis: body height entry, removal of footwear, foot electrode preparation, participant positioning, bioimpedance measurement, and automated report generation.

Estimated time: 5 minutes per participant.

Cost calculation

Average gross monthly wage in Hungary: 756,400 HUF (Hungarian Central Statistical Office, 2025).

Assuming 170 working hours per month:

Hourly wage = 4,449 HUF/hour

Cost per minute = 74.15 HUF/min

Table S6. Cost per measurement.

Method	Time (min)	Cost (HUF)	Cost (EUR)
**BMI**	2	148	0.37
**InBody**	5	371	0.94

Abbreviations: BMI, body mass index; min, minutes; HUF, Hungarian forint; EUR, euro.

Screening capacity

Screening throughput is calculated as:

Patients/hour = 60 / measurement time

Table S7. Screening capacity per hour.

Strategy	Time (min)	Patients/hour
**BMI-only**	2	30
**InBody-only**	5	12

Abbreviations: BMI, body mass index; min, minutes.

Mixed screening strategy

Based on the inflection-point analysis (Supplementary Materials S2), 51.9% of the Hungarian adult population falls into BMI ranges where InBody measurement provides additional diagnostic value.

Therefore, a mixed strategy can be defined:

48.1% BMI-only screening

51.9% BMI + InBody screening

Weighted measurement time:

$(0.481 \times 2) + (0.519 \times 5) = 3.56$ minutes

Mixed screening capacity:

60 / 3.56 = 22.7 patients/hour

Mixed screening cost:

(0.481 × 148) + (0.519 × 371) = 264 HUF per participant

Table S8. Cost and capacity comparison.

Strategy	Cost (HUF)	Cost (EUR)	Patients/hour
**BMI-only**	148	0.37	30
**Mixed BMI + InBody**	264	0.67	22.7
**InBody-only**	371	0.94	12

Abbreviations: BMI, body mass index; HUF, Hungarian forint; EUR, euro.

Population-level screening costs

Using the Hungarian adult population size of 7,921,212 adults, the total labor cost of one screening round can be estimated as follows:

Table S9. Population-level screening costs.

Strategy	Total cost (HUF)	Total cost (EUR)
**BMI-only**	1.17 billion	2.97 million
**Mixed strategy**	2.09 billion	5.30 million
**InBody-only**	2.94 billion	7.45 million

Abbreviations: BMI, body mass index; HUF, Hungarian forint; EUR, euro.

Sensitivity analysis

To evaluate robustness of the model, deterministic one-way sensitivity analyses were performed for two key parameters: hourly wage and measurement time. Each parameter was varied by ±20% to reflect plausible real-world variability.

Table S10. Wage sensitivity analysis (±20%)

Scenario	BMI (HUF)	Mixed (HUF)	InBody (HUF)
**Low wage (-20%)**	118	211	297
**Base case**	148	264	371

**High wage (+20%)**	178	317	445

Abbreviations: BMI, body mass index; HUF, Hungarian forint.

Table S11. Measurement time sensitivity (±20%)

Scenario	BMI (/hour)	Mixed (/hour)	InBody (/hour)
**Faster workflow (-20%)**	37.5	28.4	15
**Base case**	30	22.7	12
**Slower workflow (+20%)**	25	18.9	10

Interpretation

BMI-only screening provides the highest throughput and lowest cost, making it suitable for universal population screening. InBody-only screening substantially reduces screening capacity and increases labor costs. The mixed strategy offers a balanced solution, maintaining substantially higher screening capacity while requiring only about 71% of the cost of universal InBody screening. Results remained stable across all tested sensitivity scenarios.
